# Molecular Characterization of the GALC Mutation Thr112Ala Causing Krabbe Disease

**DOI:** 10.3390/ijms26178647

**Published:** 2025-09-05

**Authors:** Lukas Heger, Piet Ankermann, Eileen Socher

**Affiliations:** 1 Department of Transfusion Medicine and Hemostaseology, Universitätsklinikum Erlangen, Friedrich-Alexander-Universität Erlangen-Nürnberg (FAU), 91054 Erlangen, Germany; lukas.heger@uk-erlangen.de; 2 Institute of Functional and Clinical Anatomy, Friedrich-Alexander-Universität Erlangen-Nürnberg (FAU), 91054 Erlangen, Germany; piet.ankermann@fau.de

**Keywords:** Krabbe disease, β-galactocerebrosidase, GALC, mutations, Thr112, protein structure, homology model, molecular dynamics simulations

## Abstract

Krabbe disease is a rare and severe lysosomal disorder affecting the white matter of the central and peripheral nervous system. It is characterized by neurodegeneration, with the most common form being infantile Krabbe disease, typically diagnosed within the first year of life. This autosomal-recessive disease is caused by mutations in the *GALC* gene, which encodes the lysosomal enzyme β-galactocerebrosidase. This study focuses on a β-galactocerebrosidase variant, with Thr112Ala identified as a homozygous mutation in a patient with infantile Krabbe disease. To understand the structural effects of this mutation, we conducted all-atom molecular dynamics simulations of both the mutant and wild-type (wt) enzymes at cytosolic (pH 7.0) and lysosomal pH (pH 4.5), as β-galactocerebrosidase is localized in the lysosome. The results showed differences in protein flexibility, the hydrogen bond network, and the stability of secondary structure elements between the wild type and mutant enzymes. Additionally, the mutation affected the size of the substrate-binding pocket at lysosomal pH, even though the mutation site is not part of the active/binding site of the enzyme. These findings provide valuable insights into how the mutation impacts the structure of β-galactocerebrosidase in the lysosomal environment, contributing to the understanding of Krabbe disease’s molecular mechanisms.

## 1. Introduction

Krabbe disease, also known as globoid cell leukodystrophy or galactocerebrosidase (GALC) deficiency, is a rare, autosomal-recessive lysosomal storage disorder that predominantly affects the central and peripheral nervous systems. The disease is caused by mutations in the *GALC* gene, leading to a deficiency of the lysosomal hydrolase galactocerebrosidase (GALC). This deficiency impairs the hydrolysis of galactolipids, including β-D-galactocerebroside and psychosine, which are normally cleaved into β-D-galactose and ceramide, or β-D-galactose and sphingosine, respectively [1]. A GALC deficiency results in the accumulation of psychosine, a cytotoxic metabolite, that induces apoptosis in oligodendrocytes and Schwann cells, thereby triggering widespread demyelination [2,3,4]. Clinical manifestations typically appear within the first few months of life and include irritability, feeding difficulties, spasticity, developmental regression, and eventually, severe neurodegeneration and early mortality. Although hematopoietic stem cell transplantation can modestly delay disease progression in some pre-symptomatic patients, no curative therapy currently exists. Therefore, understanding the molecular basis of GALC dysfunction in Krabbe disease is critical for advancing both diagnostic and therapeutic approaches [5,6,7].

Over 140 mutations in the *GALC* gene, which is located on chromosome 14, have been reported to date, including missense, nonsense, frameshift, and splice site mutations [8,9,10,11,12,13]. These genetic variants can result in reduced GALC activity due to impaired protein folding, trafficking, or direct catalytic defects. Among the known missense mutations, the threonine-to-alanine substitution at position 112 (Thr112Ala) has attracted interest due to its clear impact on enzymatic function by a rather minor substitution, based on the similarity of the amino acids [14]. Nevertheless, this mutation replaces a polar threonine with a smaller, non-polar alanine, potentially altering local hydrogen bonding and stability. Although the Thr112Ala variant is frequently found in newborns screened positive for Krabbe disease and several individuals have been identified with low or borderline GALC activity, its structural and pathogenic significance remains to be fully understood [9,15,16]. In 2019, Nashabat et al. published a case report about a six-year-old female patient with a novel phenotype of infantile Krabbe disease [14]. The patient had a homozygous mutation c.334A>G (p.Thr112Ala) in the *GALC* gene and suffered from developmental delays, atypical hypopigmentation lesions, and hypoventilation [14]. It was already known from previous case reports and studies that the Thr112Ala mutation should be classified as pathogenic. However, those previously reported patients were compound heterozygous, meaning they had this mutation combined with other mutations in the *GALC* gene. To our knowledge, Nashabat et al. were the first to describe a patient with infantile Krabbe disease due to the homozygous mutation c.334A>G (p.Thr112Ala), suggesting that all aspects of her phenotype can be directly attributed to this point mutation [14]. However, the exact effect of this point mutation on the protein structure and, thus, on the enzyme function at the atomic level was still unclear. Investigating the molecular impact of this substitution could yield valuable insight into genotype–phenotype relationships and assist in variant classification [17,18].

The structure of the human 685 amino acid GALC protein has not been experimentally resolved until today. However, Deane et al. presented in 2011 the first structures of mouse GALC (83% sequence identity with human GALC) determined by X-ray crystallography at resolutions better than 2.5 Å [19]. The protein adopts a multi-domain architecture consisting of a central triosephosphate isomerase (TIM) barrel harboring the active site, flanked by a β-sandwich domain and the C-terminal lectin domain. The active site contains several residues involved in substrate binding and catalysis, including Glu274 and Glu198, which act as the catalytic nucleophile and proton donor/acceptor, respectively. The residue Thr112 is located near the N-terminal region, outside the catalytic pocket, but within a structurally conserved region that contributes to overall folding and domain stability. Given the importance of domain–domain interactions for proper enzyme function, even substitutions like Thr112Ala could have long-range effects on protein conformation or dynamics. Structural analysis of this region is therefore crucial for understanding the mutation’s pathogenic potential.

In this study, we used a combination of in silico techniques, including homology modeling, all-atom molecular dynamics (MD) simulations with protonation states corresponding to cytosolic (pH 7.0) or lysosomal pH (pH 4.5), and structural bioinformatics, to analyze the consequences of the Thr112Ala mutation on the conformation and dynamics of the GALC protein. We focused on the global effects on protein stability and flexibility, as well as local differences in flexibility, residue interactions, hydrogen bonding, secondary structure, and the volume of the substrate-binding pocket. By comparing the mutant structure to the wild-type (wt) protein based on the available X-ray crystallographic data, we aimed to characterize the structural mechanisms by which this substitution may contribute to reduced enzymatic activity. These results provide mechanistic insight into GALC misfunction in Krabbe disease and may contribute to future therapeutic strategies, such as the rational design of pharmacological chaperones that stabilize misfolded GALC variants [20].

## 2. Results

### 2.1. Homology Model of Human Galactocerebrosidase

**Figure 1:**
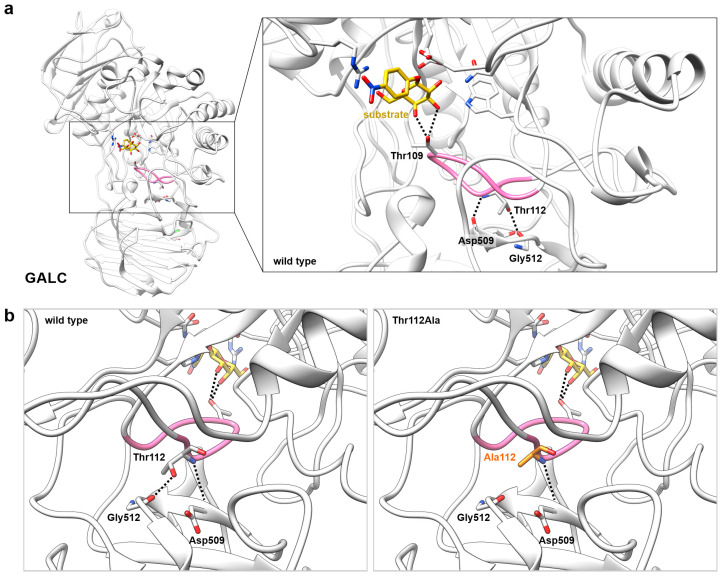
Structural representation of the GALC protein. (**a**) Human GALC protein structure with the six residues of the active/substrate binding site (including Thr109), a substrate molecule (yellow), Thr112, Asp509, and Gly512 shown as sticks. The residue Thr109 binds the substrate molecule via two hydrogen bonds (black dashed lines). Since Thr112 is located in the same loop as the substrate-binding Thr109, this loop structure was highlighted in pink. (**b**) By rotating the GALC protein, the mutation site Thr112/Ala112 is in focus. While Thr112 (left panel) can form two hydrogen bonds with the backbone atoms of Asp509 and Gly512, alanine at position 112 (right panel, orange) has only the hydrogen bond with Asp509 because alanine is not capable of forming any hydrogen bond with its side chain.

As the homozygous Thr112Ala mutation in GALC causes Krabbe disease [14], we were interested in how this single amino acid substitution reduces the enzymatic activity of GALC. To find explanatory approaches for this experimental finding on a structural level, the protein structure for human GALC was generated by homology modeling. As a template, the X-ray crystal structure of the murine GALC (PDB ID: 4CCC [1]) was selected due to its 83% sequence identity to the human GALC. The quality of the homology model was validated by Ramachandran plots. The analysis showed that almost all of the residues were in favored (95.788%) or allowed regions (3.588%, Figure S1). For the visual representation, a substrate molecule was placed in the active site (Figure 1a). To illustrate the high structural similarity between murine and human GALC, their structures were superimposed, showing an almost identical active site of both GALC structures (Figure S2a). As the Thr112Ala mutation caused Krabbe disease, we first analyzed the active site of GALC. In the magnification of the active site of the GALC protein structure (Figure 1a, right panel), as well as in the two-dimensional representation of the interactions between the substrate molecule and the amino acids belonging to the active site and the binding site (Figure S2b), respectively, it could be seen that Thr109 belongs to the binding site and interacts with the substrate via two hydrogen bonds. It is also noteworthy at this point that Thr109 interacts with a part of the substrate molecule that is not altered by the catalyzed reaction and, therefore, also plays a role in the binding of the product galactose [19]. The residue Thr112, which is mutated to alanine in some patients, was located in the same loop as the substrate-binding Thr109 and, therefore, is in a very close vicinity to the active/binding site (Figure 1). A more detailed investigation of the protein structure around residue Thr112 revealed hydrogen bonds between Thr112 and two other residues in the C-terminal lectin domain of GALC (Figure 1b, left panel). The backbone of Thr112 forms a hydrogen bond with the backbone of Asp509, while the polar side chain of Thr112 interacts via a hydrogen bond with the backbone of Gly512. The Thr112Ala mutation resulted in the loss of the hydrogen bond with Gly512 due to the shorter aliphatic side chain of alanine (Figure 1b, right panel). In summary, the Thr112 residue, which is mutated in some patients, was not directly involved in the assembly of the active site and was also not located at the protein surface, where it could be part of the interaction interface with other proteins (e.g., GALC-associated saposin SapA (PDB ID code: 5NXB [21])). However, we observed that the loss of this hydrogen bond with Gly512 caused increased flexibility in the loop around the residue at position 112, where the substrate-binding Thr109 is also located. Thereby, the mutation might have an indirect effect on the binding site and the enzymatic activity.

### 2.2. Differences in Protein Flexibility at Lysosomal pH

In order to provide a comprehensive description of the functional effects of the Thr112Ala mutation at the atomic level, it is not sufficient to analyze only the static structure. Therefore, molecular dynamics (MD) simulations were conducted. These MD simulations were performed on the GALC protein in its wild-type form and also with the Thr112Ala missense mutation. An empty substrate-binding pocket was selected to resemble the structural state prior to the entry of a substrate molecule into the binding pocket, with the objective of maximizing the flexibility of the substrate-binding pocket. The protonation of the residues in GALC was selected to match cytosolic (pH 7) or lysosomal pH (pH 4.5) in order to simulate the conditions that prevail in some experimental setups (pH 7), but more importantly, to choose the conditions that are natural for a lysosomal protein (pH 4.5) such as GALC. To enable statistical analyses, four simulation runs were performed for all four cases (wt GALC at pH 7.0 and pH 4.5, as well as GALC with Thr112Ala at pH 7.0 and pH 4.5).

**Figure 2:**
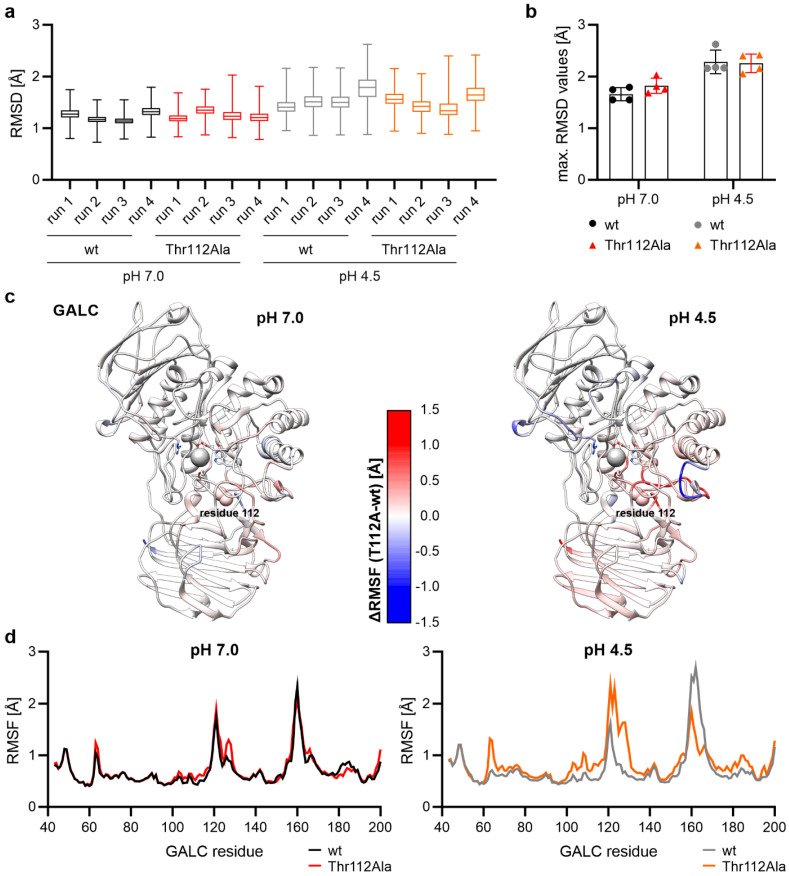
Global structural stability and flexibility of GALC. (**a**) The root-mean-square deviation (RMSD) was determined for all backbone atoms of the GALC proteins. The mean value in every simulation run was below 2 Å, and (**b**) the maximum RMSD values were all below 3 Å, indicating a high structural stability. The values are shown as (**a**) box plots (min. to max.) or (**b**) mean as bar graph ± standard deviation (SD). (**c**) The root-mean-square fluctuation (RSMF) was calculated as the difference between the mean values of the mutated GALC variant and the wild-type GALC. This difference is shown color-coded for every GALC residue at cytosolic pH (pH 7.0, left) and lysosomal pH (pH 4.5, right). (**d**) RMSF values for backbone atoms of the N-terminal residues of GALC at cytosolic and lysosomal pH.

The first step in the analysis of the 500 ns-long MD simulations was to consider the structural stability of the protein during the simulation. For this purpose, the root-mean-square deviation (RMSD) was determined for all backbone atoms of the protein. The mean value was less than 2 Å for each of the 16 simulation runs (Figure 2a), and the maximum RMSD values were all below 3 Å (Figure 2b), indicating that the protein structure was very stable during the simulations. In a second step, the fluctuation of individual amino acids in GALC was analyzed by measuring the root-mean-square fluctuation (RMSF) of the backbone atoms. When the difference between the RMSF values for the wt GALC protein and the mutant protein with Thr112Ala was calculated for the individual amino acids at pH 7, it was noticeable that this difference was close to 0 Å across the entire protein (Figure 2c, left). Only at pH 4.5, the pH at which GALC is active in the lysosome, did we observe regions in the protein that showed more or less fluctuations (Figure 2c, right). Large differences in the fluctuation of the backbone atoms were particularly evident for the first 200 N-terminal amino acids of GALC (Figure 2d). While only a few minor differences in the RMSF of the backbone atoms could be detected at cytosolic pH (pH 7), the differences at lysosomal pH (pH 4.5) were significant. Especially around the mutation site at position 112, and also around Tyr129, a residue involved in a water-mediated interaction with Thr112 in the crystal structure of murine GALC, a larger fluctuation could be observed. In contrast, a lower degree of fluctuation was exhibited for the Thr112Ala variant in the region encompassing residues 160–170. At the end, differences in protein flexibility between the wild type and the mutant enzyme were more prominent at lysosomal than at cytosolic pH.

### 2.3. Thr112Ala: Higher Local Flexibility in the Vicinity Around Residue 112

**Figure 3:**
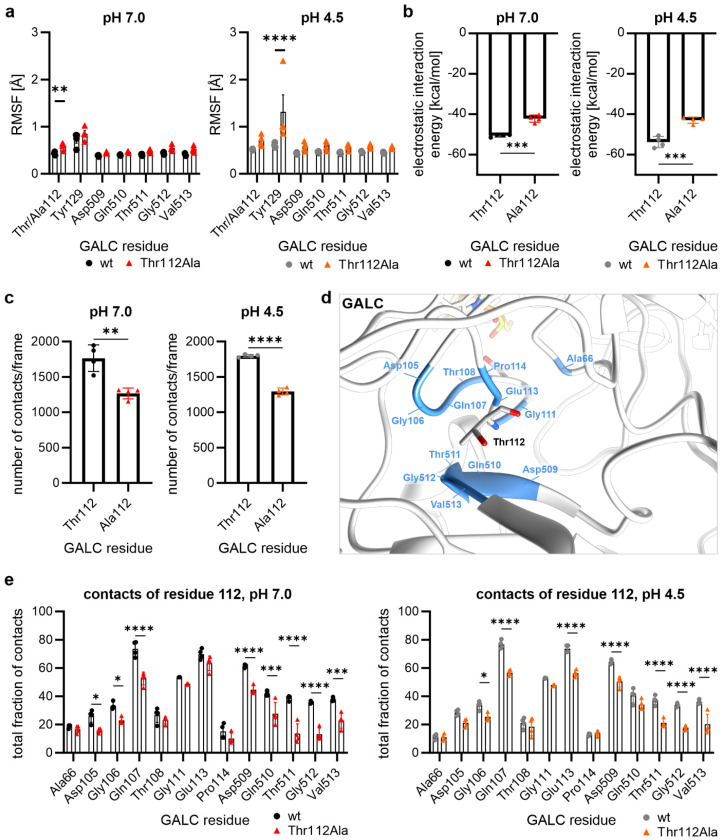
Local flexibility and interactions around GALC residue 112. (**a**) RMSF values of Thr112 or Ala112 and residues in close proximity. (**b**) Electrostatic linear interaction energies of residue 112 in wt (black/gray) or the Thr112Ala variant (red/orange) of GALC. (**c**) The number of contacts formed by Thr112/Ala112 with all other residues in GALC. (**d**) Structural representation of the residues in close contact with the residue at position 112. (**e**) Residue-specific analysis of the number of contacts between Thr or Ala at position 112 and their interaction partners within GALC. (**a**–**c**,**e**) Mean values are shown as bar graphs ± SD (n = 4). Statistical testing was performed in GraphPad Prism (V10) using (**a**,**e**) 2-way ANOVA with Šídák’s multiple comparisons test or (**b**,**c**) unpaired t test (* *p* < 0.05, ** *p* < 0.01, *** *p* < 0.001, **** *p* < 0.0001).

Analyzing the RMSF values for the individual amino acids in the vicinity of the mutation site at position 112, the GALC mutant Thr112Ala showed significantly higher RMSF values for Thr112/Ala112 at cytosolic and Tyr129 at lysosomal pH (Figure 3a). In order to understand the differences around the mutation site at position 112 in detail, further analyses based on the amino acid Thr112 or Ala112 were necessary. Analysis of the electrostatic interaction energy showed that alanine at position 112 exhibits diminished levels of electrostatic interactions compared to Thr112 at both cytosolic and lysosomal pH due to its non-polar side chain (Figure 3b), which also causes the loss of the hydrogen bond with Gly512 due to the missing hydroxyl group (cf. Figure 1b).

As the homology model showed fewer interactions of Ala112 compared to Thr112 (Figure 1b), we hypothesized that a reduced number of contacts in the Thr112Ala variant was also responsible for the higher flexibility of the protein at lysosomal pH (pH 4.5) (Figure 2d and Figure 3a). At cytosolic and, also, at lysosomal pH, we observed that the alanine at position 112 had fewer contacts with other amino acids from GALC (distance criterion: 5 Å; Figure 3c). At this point, it could be assumed that this is due to differences in side chain interactions. To test this, we analyzed with which other amino acids from GALC the Thr112 in the wild type or the Ala112 in the mutant GALC variant interacts (Figure 3d). Only interactions that lasted at least 5% of the simulation time (at least 25 ns) were included in this analysis. This revealed differences in the number of contacts with the residues immediately adjacent to the mutation site (Asp105 to Pro114), as well as much more significant differences with the region Asp509 to Val513 (Figure 3e). Thus, the Thr112Ala mutation led to enhanced protein flexibility due to fewer contacts of the non-polar alanine side chain.

### 2.4. Hydrogen Bonds of the Residue at Position 112

Hydrogen bonds between the different amino acids of a protein belong to the category of structure-stabilizing interactions. In principle, amino acids within a protein can form hydrogen bonds with their backbone atoms or, if present, with their polar side chains (e.g., the hydroxyl group in the threonine side chain). The analysis of intramolecular hydrogen bonds of a mutated residue can, therefore, be divided into two groups: One group involves hydrogen bonds in which the backbone atoms of the respective amino acids are involved, while the second group contains hydrogen bonds in which the atoms of the side chain of the mutated amino acids are involved.

**Figure 4:**
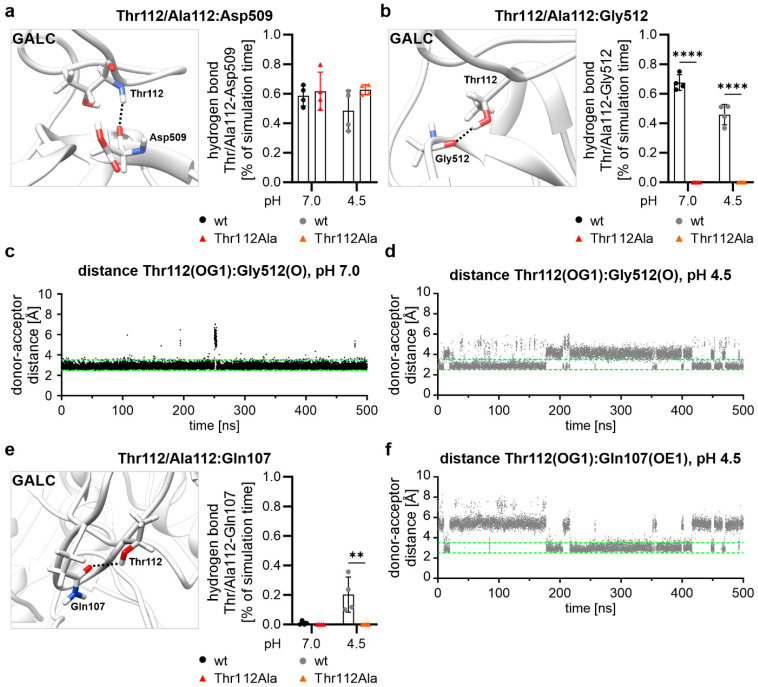
Hydrogen bonds of Thr/Ala112. (**a**) Structural representation of the hydrogen bond (indicated by a black dashed line) between the backbone atoms of Thr112 and Asp509. Asp509 is shown with protonated side chain. Hydrogen bond occurrence was measured for each simulation run of the wild type and the GALC variant with Thr112Ala and at each pH value. (**b**) Structural representation of the hydrogen bond (indicated by a black dashed line) between the hydroxyl group of Thr112 and Gly512. Hydrogen bond occurrence was measured for each simulation run of the wild type and the Thr112Ala variant at both pH values. The GALC variant with Thr112Ala is not able to form this hydrogen bond due to the non-polar alanine side chain. (**c**,**d**) Time-resolved distance plot for simulation run 1 describes the distance between the donor and acceptor atoms of the atoms of Thr112 and Gly512 in the wild type at pH 7.0 (**c**) or at pH 4.5 (**d**) (green dashed lines: distances within these values are defined as hydrogen bonds). (**e**) Structural representation of the hydrogen bond (indicated by a black dashed line) between the hydroxyl group of Thr112 and the side chain of Gln107. Hydrogen bond occurrence was measured for each simulation run of the wild type and the Thr112Ala variant at both pH values. Due to the non-polar alanine side chain, the GALC variant with Thr112Ala is not able to form this hydrogen bond. (**f**) Time-resolved distance plot for GALC wild-type simulation run 1 at pH 4.5 describes the donor–acceptor distance of the Thr112:Gln107 hydrogen bond over the simulation time (green dashed lines: distances within these values are defined as hydrogen bonds). (**a**,**b**,**e**) Mean values ± SD are shown as bar graphs (n = 4). Statistical testing was performed in GraphPad Prism (V10) using 2-way ANOVA with Šídák’s multiple comparisons test (** *p* < 0.01, **** *p* < 0.0001).

When investigating which hydrogen bonds Thr112 or Ala112 form, we started with the hydrogen bonds formed by the backbone atoms of Thr112 and Ala112. It was observed that these atoms form only a hydrogen bond with the backbone atoms of Asp509. The analysis of the occurrence of this hydrogen bond over the simulation time (i.e., reflecting the stability of the hydrogen bond) revealed no significant differences between the wild-type GALC and the mutated variant (Figure 4a). This observation is consistent with the fact that the Thr112Ala mutation exclusively changes the side-chain atoms at position 112, leaving the backbone atoms unaltered.

However, mutating Thr112 to alanine causes the loss of the hydrogen bond that Thr112 forms between its side-chain hydroxyl group and the backbone of Gly512 (Figure 4b, cf. Figure 1b). This occurs because alanine has a non-polar side chain, which is incapable of forming hydrogen bonds. Moreover, analysis of the Thr112–Gly512 hydrogen bond demonstrated that, even in the wild type, this hydrogen bond was less stable at lysosomal pH than at cytosolic pH (Figure 4b–d and Figure S3a,b), which is likely attributable to the elevated fluctuation at lysosomal pH and potentially to alterations in the surrounding electrostatic field caused by different protonation states. The analysis of the Thr112-Gly512 hydrogen bond (Figure 4b) utilized both a distance criterion and an angle criterion for the definition of the hydrogen bond, revealing no values exceeding 80% of the simulation time. Conversely, a hydrogen bond could be postulated for the wild type at cytosolic pH for almost the whole simulation, when only the distance between the donor and acceptor atom (Figure 4c and Figure S3a) was taken into account. At lysosomal pH, however, it became evident that the hydrogen bond was more unstable, also when only the donor–acceptor distance was taken into consideration (Figure 4d and Figure S3b). Thus, the Thr112–Gly512 hydrogen bond undergoes a process of breakdown, followed by recurrent formation. In the phases of the MD simulation in which Thr112 does not form a hydrogen bond with Gly512 at lysosomal pH, we observed that the Thr112 side chain often rotates and establishes a hydrogen bond via its hydroxyl group with the side chain of Gln107 (Figure 4e,f and Figure S3c). As this interaction is not possible with the non-polar side chain of alanine, our results suggest that the polar side chain of Thr112 is necessary for the enhanced stability at lysosomal pH around the mutation site (Figure 2c).

### 2.5. Influence of the Mutation Thr112Ala on Substrate-Binding Residues and Residues of the Active Site

**Figure 5:**
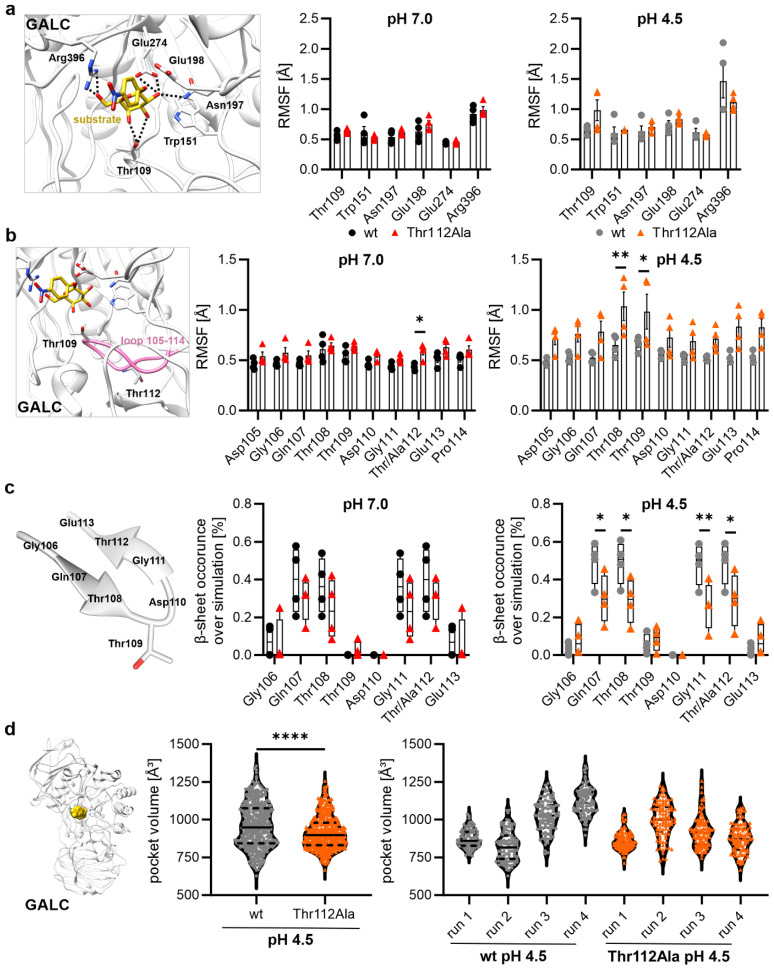
Substrate-binding pocket of GALC. (**a**) Structural representation and RMSF values of the substrate-binding residues (Thr109, Trp151, Asn197, and Arg396) and active site residues (Glu198 and Glu274) at neutral pH (black/red) or lysosomal pH value (gray/orange). (**b**) Structural representation and RMSF values of the loop region Asp105 to Pro114 at neutral pH (black/red) and lysosomal pH value (gray/orange). (**c**) Structural representation of the β-hairpin Gly106-Gly113 and β-sheet occurrence of the region Gly106 to Glu113 at cytosolic pH (black/red) or lysosomal pH value (gray/orange). Values of the single runs are shown as box plots (min. to max. with mean as horizontal line). (**d**) Structural representation of GALC with a substrate (golden color) and the volume of the substrate-binding pocket at lysosomal pH (summarized over all four simulation runs (middle) and for each individual run (right)). (**a**,**b**) Mean values ± SD are shown as bar graphs (n = 4). Statistical testing was performed in GraphPad Prism (V10) using (**a**–**c**) 2-way ANOVA with Šídák’s multiple comparisons test or (**d**) unpaired t test (* *p* < 0.05, ** *p* < 0.01, **** *p* < 0.0001).

There are various ways in which mutations can influence the activity of enzymes. For instance, a mutation can modify the activity of an enzyme by altering the binding pocket of the substrate. Therefore, the subsequent objective was to ascertain whether the MD simulations also demonstrate changes in the substrate-binding pocket. The fluctuation analysis of substrate-binding residues (Thr109, Trp151, Asn197, and Arg396) and active site residues (Glu198 and Glu274) did not reveal any significant differences (Figure 5a). However, the substrate-binding residue Thr109 showed a higher flexibility at lysosomal pH (pH 4.5) in the Thr112Ala mutant (Figure 5a,b).

The residue Thr109 is located in close proximity to Thr112 within a flexible region (see Figure 1 and Figure 5b). A detailed analysis of the fluctuation of this entire loop region, Asp105 to Pro114, clearly demonstrated that this loop region exhibits increased flexibility in the mutant variant at cytosolic and lysosomal pH in comparison to the wild-type GALC (Figure 5b). Visual inspection of the trajectories of the MD simulations provided evidence that these changes in flexibility were related to the occurrence of a β-hairpin (a secondary structure formed by two antiparallel β-sheet strands with only two intervening residues forming a turn between them) in this loop (Figure 5c). Therefore, we determined the time that the residues Gln106 to Glu113 participated in the β-sheet strands of the observed β-hairpin. Notably, the mutant GALC variant exhibited at both pH values a reduced frequency of beta-sheet formation during the MD simulation when compared to the GALC wild type. This effect could be detected to a greater extent at lysosomal pH (pH 4.5) in comparison with cytosolic pH (pH 7.0) due to a more stable β-hairpin structure in wild-type GALC at lysosomal pH (e.g., through the formation of a hydrogen bond between Thr112 and Gln107). As this β-hairpin is not present in the crystal structure of murine GALC (PDB: 4CCC [1]), this seems to be a transient structural element stabilizing the structure of GALC.

### 2.6. Mutational Effect on the Size of the Binding Pocket at pH 4.5

In the final step, the general geometry of the substrate-binding pocket was therefore also analyzed at lysosomal pH by measuring the size of the binding pocket for 100 snapshots evenly distributed over each of the simulations at cytosolic pH (Figure 5d). Overall, we observed a significantly reduced size of the binding pocket in the Thr112Ala mutant compared to the wild-type GALC (wild-type: 959.82 ± 148.10 Å^3^ vs. GALC with Thr112Ala: 913.29 ± 108.77 Å^3^). In the wild-type GALC, the volume of the binding pocket fluctuated between 500 and 1500 Å^3^, demonstrating broad flexibility necessary for entry and release of the substrate and the product of GALC, respectively (Figure 5d). In contrast, the Thr112Ala mutant was less flexible with a pocket volume of 800 to 1000 Å^3^ over the majority of the simulation time (Figure 5d). This reduced flexibility might hinder the entry of the substrate molecule into the binding pocket, contributing to the reduced activity of the mutant GALC protein. We then analyzed the individual runs of the simulation to determine whether we observed the larger changes in the binding pocket in all simulations. This analysis revealed that, in two out of four simulation runs for the wild-type GALC, the mean volume of the binding pocket exhibited a tendency to decreased values, while in the remaining two simulation runs, a tendency towards an increased mean volume was observed. This finding suggests that the binding pocket in the wild type could adopt an open conformation over a prolonged period of time, although not in every single simulation run. In contrast, the MD simulations of the GALC variant with Thr112Ala did not demonstrate this broad spectrum in the mean volume of the binding pocket at lysosomal pH. Instead, all four simulations of the mutated GALC showed a reduced volume of the binding pocket (volume > 1000 Å^3^: wild type: 39.75% of the analyzed snapshots; Thr112Ala: 20.00% of the analyzed snapshots). Thus, the GALC variant Thr112Ala might have lost the ‘breathing’ motion of the binding pocket necessary for proper enzymatic activity of GALC. In order to analyze whether the Thr112Ala mutation has an impact on the internal motions of GALC, we performed a dynamic cross-correlation analysis of the wild-type GALC and the Thr112Ala variant at lysosomal pH (Figure S4). However, the general motions of the different protein domains of GALC were not influenced by the Thr112Ala mutation (Figure S4). In summary, the effects of the mutation Thr112Ala on the volume of the binding pocket could be observed at lysosomal pH, although the mutation site itself was not part of the active site/binding site of the enzyme.

## 3. Discussion

Krabbe disease belongs to the group of lysosomal storage disorders and is caused by mutations in the *GALC* gene, leading to a deficiency of the lysosomal hydrolase galactocerebrosidase (GALC) and subsequently, to an accumulation of psychosine—a cytotoxic metabolite that induces apoptosis in oligodendrocytes and Schwann cells. Over 140 mutations in the *GALC* gene are known, but it is often unclear how the single mutations affect GALC activity and, thus, whether they are causative for the phenotype of the disease. Here, we used a combination of in silico techniques, including homology modeling, all-atom molecular dynamics (MD) simulations with protonation states corresponding to cytosolic (pH 7.0) or lysosomal pH (pH 4.5), and structural bioinformatics, to analyze the consequences of the Thr112Ala mutation on the conformation and dynamics of the GALC protein. We demonstrate that the mutation affects protein flexibility, the hydrogen bond network, and the stability of secondary structure elements of GALC, as well as the accessibility of the substrate-binding pocket at lysosomal pH. Thereby, we provide a molecular explanation for the pathogenic nature of the Thr112Ala mutation, which might aid in the diagnosis of patients with compound heterozygous mutations and allow for the development of new therapeutic approaches.

Several case reports of compound heterozygote patients with late-onset Krabbe disease, as well as one homozygous patient with infantile Krabbe disease harboring the Thr112Ala variant, suggested a pathogenic nature of this mutation [14,15,16,22]. Further, the variant is frequently found in newborns screened positive for Krabbe disease, whereas the prevalence of the mutation is low in healthy controls [9,16]. Moreover, it was demonstrated in fibroblasts, as well as in leukocytes, from patients with the Thr112Ala variant that the enzymatic activity of GALC is reduced and thereby can lead to an infantile onset of Krabbe disease [14,22]. However, it was unclear how the mutation affects the activity of GALC, as it is neither involved in substrate binding nor in enzymatic function [1]. Our results suggest that the region containing the substrate-binding residue Thr109 exhibits a higher degree of flexibility in the GALC variant with Thr112Ala, potentially influencing the entry of the substrate molecule into the substrate-binding pocket or its overall size. Additionally, it can be assumed that the mutated GALC variant exhibits diminished substrate-binding affinity due to the augmented flexibility in the loop Asp105 to Pro114. Thus, the Thr112Ala mutation seems to have an influence on the size and, therefore, also, the geometry of the binding pocket at lysosomal pH, which leads to a change in enzymatic activity because a conformation that allows the substrate molecule to enter the pocket, or the products to exit the pocket, may be adopted less frequently. This reduces the substrate affinity of the enzyme, explaining on an atomic level how the Thr112Ala mutation in GALC causes Krabbe disease. However, it should be noted that this is merely one of several potential mechanisms through which the functionality of GALC can be reduced. For the degradation of sphingolipids, the interaction of GALC with saponin A is necessary [21]. Therefore, mutations affecting the binding of GALC to saposin A might also cause Krabbe disease. However, as the Thr112Ala mutation is not located directly at the predicted saposin A interface [21], we can exclude this possibility. This suggested mode of action of the Thr112Ala mutation is different compared to other known mutations causing Krabbe disease: The mutation D528N has a minor influence on the enzymatic activity but leads to misfolding of GALC due to a second N-glycosylation side [23]. Thereby, it is not transported to the lysosome. Similarly, the L629R variant abrogates the transport to the Golgi apparatus and retains the mutated GALC in the endoplasmic reticulum [23]. Thus, not only mutations directly affecting the enzymatic activity of GALC cause Krabbe disease but also mutations affecting the localization.

Patients diagnosed with Krabbe disease often harbor compound heterozygous mutations in the *GALC* gene with unknown influence on GALC activity [24,25,26,27,28]. While it is possible to isolate cells of the patients to measure the activity of GALC to verify the diagnosis, the reduced GALC activity cannot be attributed to a specific mutation in compound heterozygous patients. This would require cloning of the single mutations, overexpression in cell lines, and in vitro experiments, which are labor-intensive, as well as time-consuming, and might result in artificial results. Further, reduced GALC activity does not explain how the mutation affects the function of GALC on an atomic level, except for the mutation of residues directly involved in the catalytic domain. Therefore, computer-based studies of protein structure at the atomic level are a valuable complement to experiments. In our study, we could show that the methods are not only suitable to identify significant differences caused by the mutation but also confirm the importance of the pH value at which the protein structures are examined. For proteins that have their activity optimized at a slightly acidic pH (e.g., pH 4.5 in the lysosome), it is not sufficient to perform enzyme activity analyses at neutral pH, as the change in protonation of the side chains caused by the lower pH has a measurable effect on the protein structure.

Using wild-type GALC, we were able to demonstrate this pH-dependent behavior at the atomic level for the hydrogen bonds Thr112–Gly512 and Thr112–Gln107. The former is less stable at lysosomal pH than at neutral pH, whereas the latter only forms at a lower pH. This suggests that, due to the different protonation states of the side chains of the surrounding acidic amino acids and histidines at neutral and lysosomal pH and the resulting changes in electrical partial charges, the hydrogen bond network within the protein forms differently. This detail is often difficult to deduce from experimentally determined X-ray crystal structures, as it is challenging to obtain protein crystals at different pH values. As lysosomal storage disorders are a large family of diseases caused by mutations in lysosomal proteins, our approach might also aid in identifying pathogenic mutations in patients with other diseases, e.g., Gaucher disease, Niemann–Pick disease, and Anderson–Fabry disease [29,30,31,32].

Based on our results, it could also be possible to design drugs for the treatment of specific mutations causing Krabbe disease. There are X-ray crystal structures of murine GALC in complex with the bound inhibitor galacto-noeurostegine at the active site at neutral and lysosomal pH [33]. A notable aspect of these structures is that they reveal a potential allosteric binding site, suggesting the possibility of designing pharmacological chaperones that target this site [33]. The exact nature of this site remained unclear, and further investigation is required [33]. Many years earlier, in 2010, Lee et al. had already identified the relatively weak inhibitor of GALC α-lobeline to rescue the function of the D528N GALC mutant and suggested that α-lobeline has the ability to function as a pharmacological chaperone to rescue the function of this mutant [23]. Hence, this could provide a useful starting point for developing therapeutically applicable GALC activators. For the functionally and structurally closely related lysosomal enzyme β-glucocerebrosidase, activators that bind to the protein surface outside the active site and increase enzymatic activity already exist [34]. Our mutation study also enabled us to identify regions of the protein that exhibit altered dynamics due to the mutation, and these regions could represent target sites for allosteric activators.

In future studies, we will address the question of whether the observed effects are unique to the Thr112Ala mutation or if other pathogenic mutations may have a similar effect on protein structure. Since known pathogenic mutations extend across all protein domains, it is also conceivable that mutations could be categorized based on their structural effects. It may even be possible to predict the structural effects of newly discovered mutations and assess the activity of the enzyme. This would aid in the diagnosis of compound heterozygous patients with Krabbe disease.

We therefore believe that our study, which identified Thr109 and the neighboring residues in the loop structure as important key residues for the activity of the GALC enzyme, will also help to identify druggable sites in the protein structure of GALC and thus contribute to the development of allosteric activators for Krabbe disease.

## 4. Materials and Methods

### 4.1. Homology Modeling of Human β-Galactocerebrosidase

The crystal structure of murine β-galactocerebrosidase complexed with 4NBDG (enzyme–substrate complex; PDB ID code: 4CCC [1]) was selected as a template for the homology modeling of the human β-galactocerebrosidase (GALC) because the sequence alignment of human GALC using the sequence taken from UniProt (accession code: P54803) and the murine GALC (UniProt accession code: P54818) showed a sequence identity of 83%. The homology model was created with SWISS-MODEL (https://swissmodel.expasy.org/), and the 2D ligand interaction diagram for the GALC–substrate interaction was generated using the academic version of Maestro 13.6 (Schrödinger Release 2023-2: Maestro, Schrödinger, LLC, New York, NY, USA, 2023). Quality of the homology model was analyzed using Ramplot, (https://www.ramplot.in/, accessed on 21 July 2025). All other protein structure visualizations of the modeled human GALC were made with UCSF Chimera (version: 1.18; [35]).

### 4.2. Generation of the Starting Structures

In order to investigate the molecular dynamics of the GALC protein with the Thr112Ala mutation, the result of the homology modelling of the wild-type GALC was taken, and the amino acid substitution Thr112Ala was introduced with the software Swiss-PdbViewer 4.1.0 [36]. The protonation states of the titratable side chains within the GALC wild type and the mutated variant were defined for pH 7.0 and pH 4.5 by using the “PQR” output files generated by the APBS-PDB2PQR software suite [37] (https://server.poissonboltzmann.org/).

### 4.3. Molecular Dynamics Simulations and Their Subsequent Analyses

The molecular dynamics simulations were performed very similar to that described before [38,39,40,41].

The Amber Molecular Dynamics software package (ambermd.org) in version 20 [42], the ff14SB force field [43] and the Amber Tool LEaP were utilized to electrically neutralize all four of the different starting structures with sodium (Na^+^) or chloride (Cl^−^) ions and solvate them with TIP3P [44] water molecules. The GALC structure was solvated in a water box with the shape of a truncated octahedron and a distance of at least 25 Å from the borders to the solute.

To ensure a smooth and physically realistic relaxation of the system prior to production simulations, a stepwise minimization scheme was used. In detail, the geometry of the initial structures was optimized through a three-stage minimization process, which results in more stable trajectories and consistent energetic convergence. In the first minimization part, all water molecules were minimized, while all other atoms were restrained at their initial positions by using a constant force of 10 kcal·mol^−1^·Å^−2^. In the second part, additional relaxation was permitted for the ions and the hydrogen atoms of the protein with an initial minimization step length of 0.001, while the remaining protein was restrained with 10 kcal·mol^−1^·Å^−2^. The third part involved the minimization of the entire protein, ions, and water molecules without any restraints. The three minimization parts always started with 2500 steps using the steepest descent algorithm, followed by 2500 steps of conjugate gradient minimization. Following minimization, the systems were equilibrated in two successive steps. In the initial step, the temperature was elevated from 10 to 310 K within 0.1 ns, with the protein being restrained by a constant force of 5 kcal·mol^−1^·Å^−2^. The second step, which had a duration of 0.4 ns, had a constant force of 5 kcal·mol^−1^·Å^−2^ for restraining only the Cα atoms of the protein. In both equilibration steps, the time step was set to 2 fs. The minimization and equilibration steps were executed on central processing units (CPUs), while the subsequent production runs were performed using pmemd.CUDA on Nvidia A100 graphics processing units (GPUs) [45,46,47]. Subsequent 500 ns-long production runs using the Particle Mesh Ewald approach were performed without any restraints and at 310 K (controlled by a Berendsen thermostat [48]). Furthermore, the constant-pressure periodic boundary conditions with an average pressure of 1 bar and isotropic position scaling were used. For bonds involving hydrogen, the SHAKE algorithm [49] was used in the equilibration and production phases. For the purpose of statistical analysis, a total of four independent 500 ns-long MD simulation runs were conducted at the pH values 7.0 and 4.5 for both the wild-type GALC and the mutated GALC.

For the trajectory analysis, the root-mean-square fluctuations (RMSF) and the analysis of contacts were performed (with a distance criterion of ≤5 Å between any pair of atoms; total fraction of contacts for residue pairs) utilizing the Amber tool cpptraj. The measurement of interatomic distances, the calculation of electrostatic linear interaction energy, the analyses of the secondary structure, the analyses of hydrogen bonds, and the dynamic cross-correlation maps were also performed using the Amber tool cpptraj (AmberTools24) [50]. In order to determine the size of the binding pocket, the volume of the binding pocket was measured for 100 snapshots of each simulation run at pH 4.5 from wild-type GALC and the mutated GALC using the POVME 3.0 (POcket Volume MEasurer) tool.

## 5. Conclusions

Krabbe disease is a rare and severe lysosomal disorder affecting the white matter of the central and peripheral nervous system. This autosomal-recessive disease is caused by mutations in the *GALC* gene, which encodes the lysosomal enzyme β-galactocerebrosidase (GALC). This study focuses on the Thr112Ala variant of GALC. We conducted all-atom molecular dynamics simulations of the mutant and wild-type GALC. Differences in protein flexibility, the hydrogen bond network, and the stability of secondary structure elements between the wild-type GALC protein and the mutant form could be detected. Additionally, the mutation affected the size of the substrate-binding pocket at pH 4.5, even though the mutation site is not part of the active/binding site of the enzyme. It can, therefore, be speculated that this influence on the size and, therefore, also the geometry of the binding pocket at lysosomal pH leads to a change in enzymatic activity because a conformation that allows the substrate molecule to enter the pocket, or the products to exit the pocket, may be adopted less frequently. This might affect the substrate affinity of the enzyme, which would result in reduced activity, explaining on a molecular level how the Thr112Ala mutation in GALC causes Krabbe disease. These findings provide valuable insights into how this mutation impacts the structure of β-galactocerebrosidase in the lysosomal environment, contributing to the understanding of Krabbe disease’s molecular mechanisms.

## Data Availability

The raw data supporting the conclusions of this article will be made available by the authors on request.

## Supplementary Materials

The following supporting information can be downloaded at https://www.mdpi.com/article/10.3390/ijms26178647/s1.

## Abbreviations

The following abbreviations are used in this manuscript:

CPU	Central Processing Unit
GALC	β-galactocerebrosidase
GPU	Graphics Processing Unit
MD	Molecular Dynamics
RMSD	Root-Mean-Square Deviation
RMSF	Root-Mean-Square Fluctuation
SD	Standard Deviation
wt	Wild Type
