# Supplementary material for: Molecular Characterization of the GALC Mutation Thr112Ala Causing Krabbe Disease
Source: Int J Mol Sci. 2025 Sep 5;26(17):8647. doi: 10.3390/ijms26178647 (PMC12429000; doi:10.3390/ijms26178647)
Supplement: Supplementary file 1 [file ijms-26-08647-s001.zip › ijms-3746538-supplementary.pdf]

Molecular Characterization of the GALC Mutation Thr112Ala Causing Krabbe Disease

Lukas Heger [1], Piet Ankermann [2] and Eileen Socher [2,*]

[1] Department of Transfusion Medicine and Hemostaseology, Universitätsklinikum Erlangen, Friedrich-Alexander-Universität Erlangen-Nürnberg (FAU), Erlangen, Germany; lukas.heger@uk-erlangen.de
[2] Institute of Functional and Clinical Anatomy, Friedrich-Alexander-Universität Erlangen-Nürnberg (FAU), Erlangen, Germany; piet.ankermann@fau.de; eileen.socher@fau.de
* Correspondence: eileen.socher@fau.de;

Supplementary data

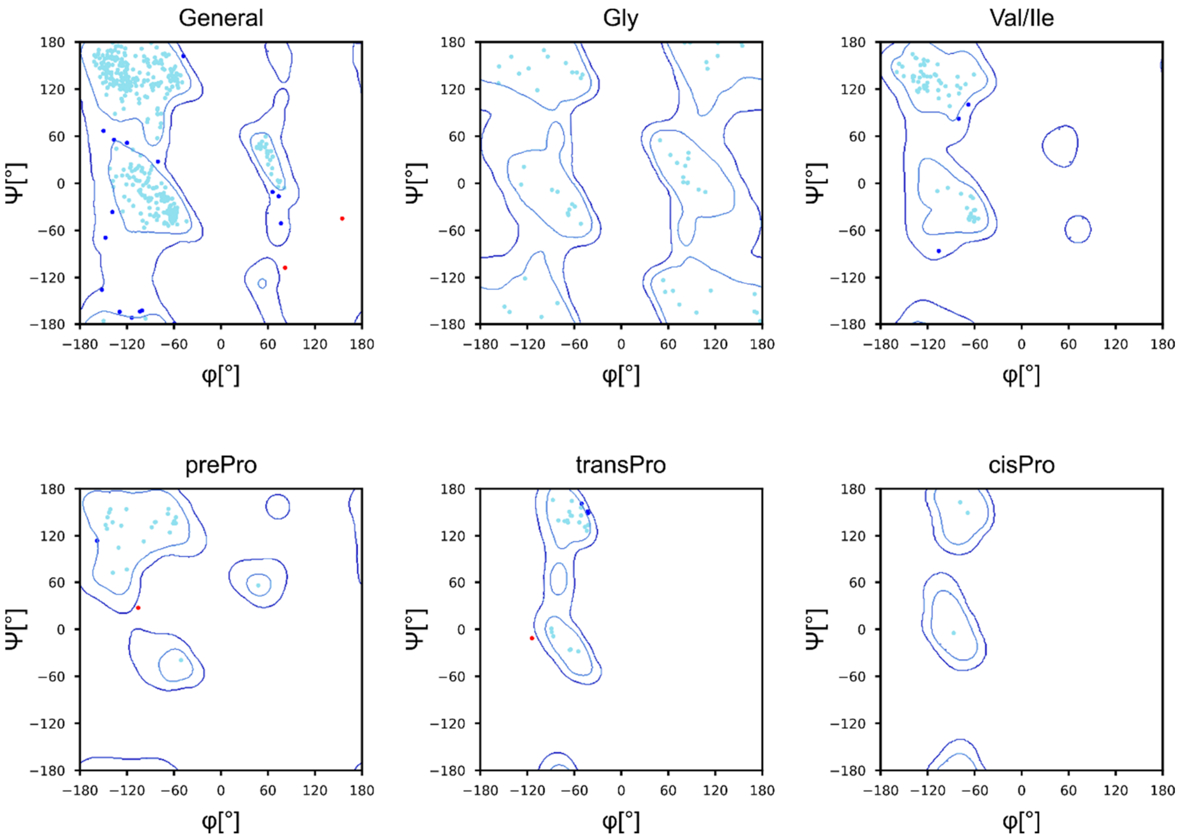

Figure S1. Ramachandran plots depict backbone dihedral angles of the homology model of human GALC. Ramachandran plots show the torsion angles of almost all amino acids (General), glycine (Gly), valine and isoleucine (Val/Ile), residues before a proline (prePro), proline with trans peptide bonds (transPro), and proline with cis peptide bonds (cisPro). Cyan dots indicates residues in favored regions and blue dots in allowed regions. Only 4 residues have backbone dihedral angles in disallowed regions (red dots).

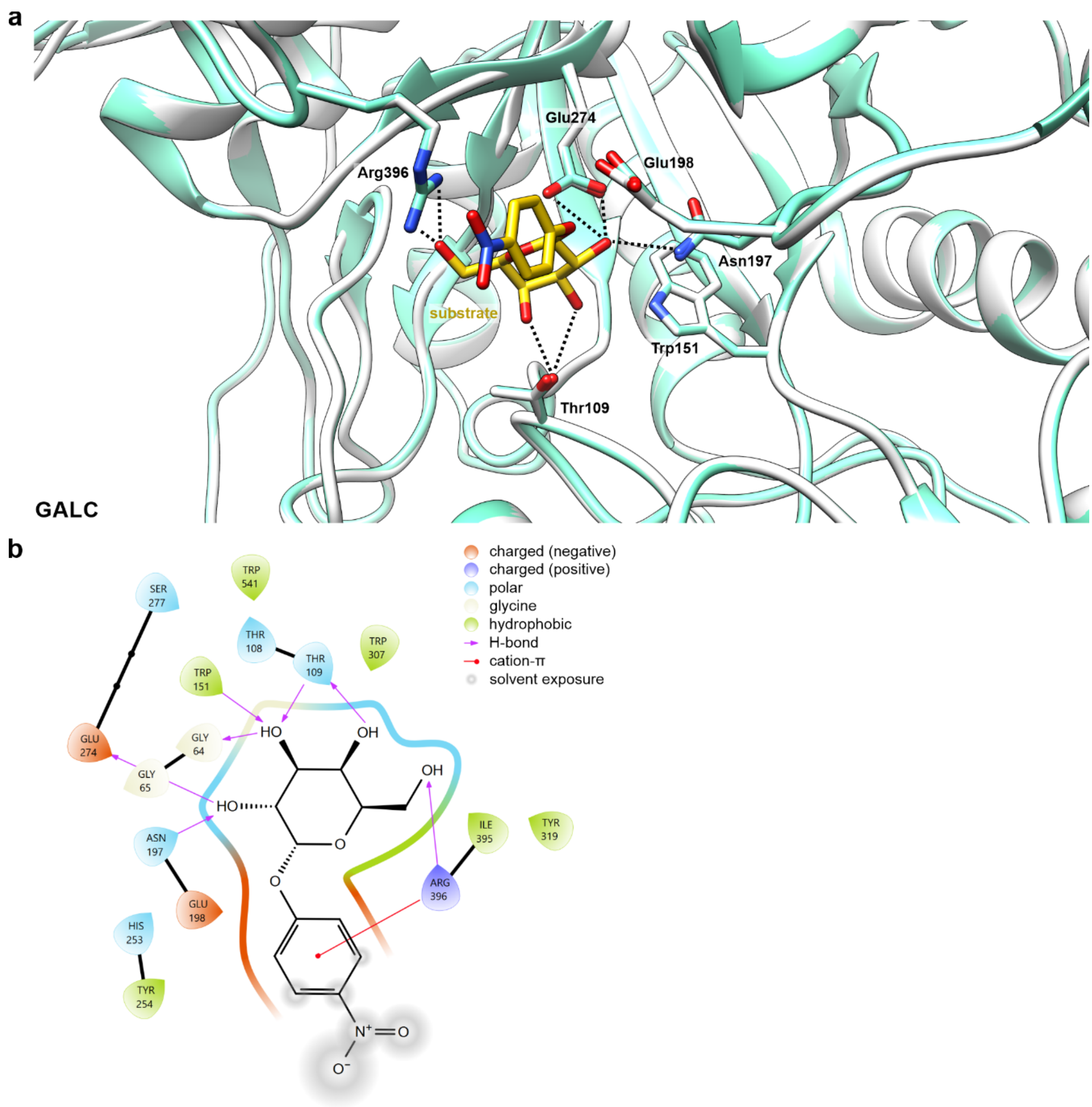

Figure S2. Superimposition of murine and human GALC and 2-dimensional representation of the interactions between the substrate and the GALC residues belonging to the active site and the binding site. (**a**) Human GALC was modeled and the substrate 4-nitrophenyl beta-D-galactopyranoside was placed into the substrate binding pocket in the same way as in the crystal structure of murine GALC (shown in aquamarine, PDB ID code: 4CCC [1]), in order to identify the GALC amino acids involved in assembling the pocket and/or interacting with the substrate. Black dashed lines represent hydrogen bonds. (**b**) The Thr109 residue, which is involved in substrate binding, interacts with the substrate via two hydrogen bonds. The residues Trp151, Asn197 and Arg396 interact with the substrate via one hydrogen bond each. Arg396 also forms a cation-π interaction with the substrate through its positively charged side chain. The two negatively charged residues of the active site, Glu198 and Glu274, appear to be further away, but this is deceptive; the three-dimensional space has been reduced to a two-dimensional plane for this type of representation.

Reference

1. Hill, C.H.; Graham, S.C.; Read, R.J.; Deane, J.E. Structural snapshots illustrate the catalytic cycle of β-galactocerebrosidase, the defective enzyme in Krabbe disease. Proc. Natl. Acad. Sci. U. S. A. 2013, 110, 20479–20484, doi:10.1073/pnas.1311990110.

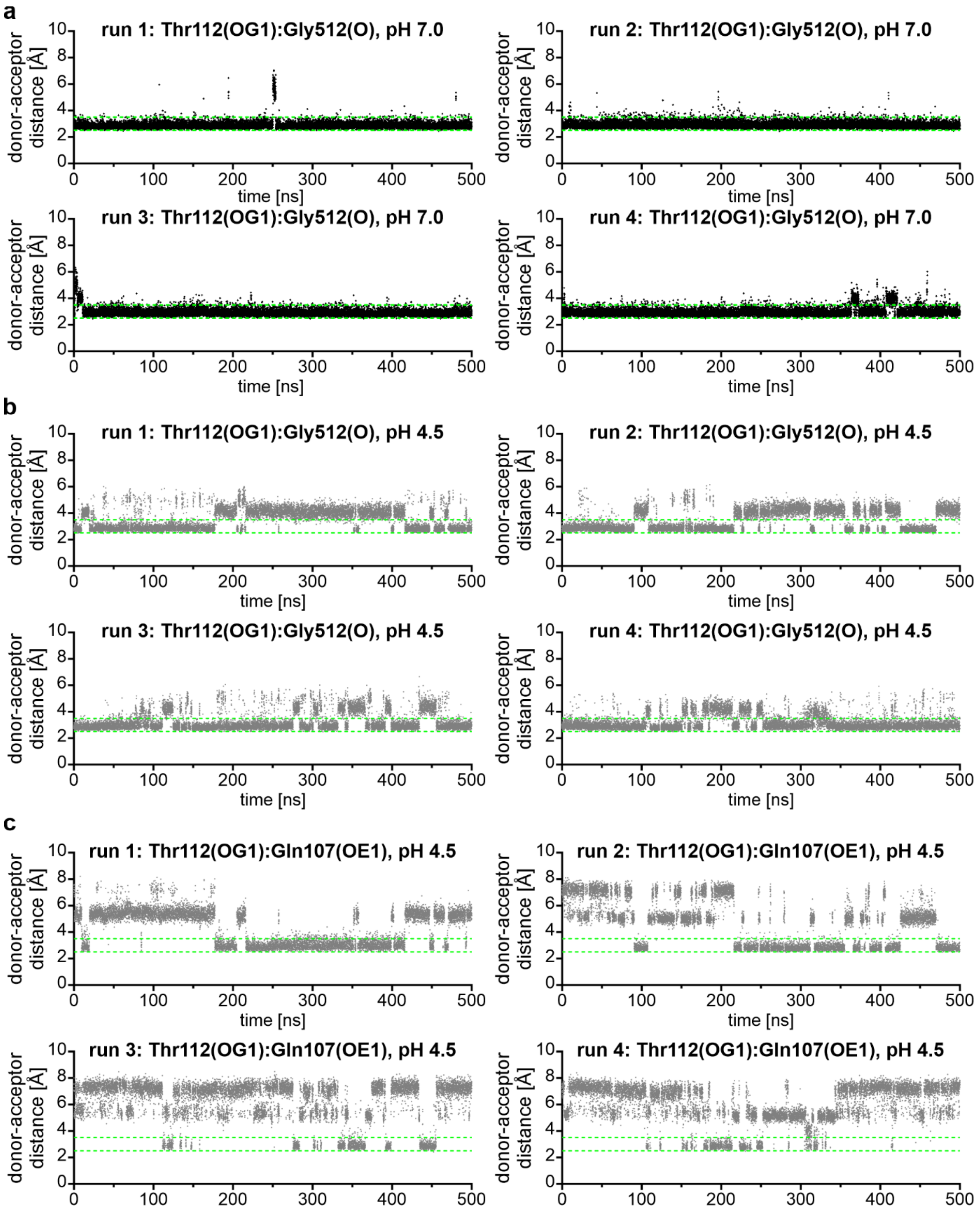

Figure S3. Hydrogen bonds of Thr112 in wild type GALC at pH 7.0 and pH 4.5. (**a**) Time resolved distance plots for every simulation run in order to describe the distance between the donor and acceptor atoms of the Thr112:Gly512 hydrogen bond in the wild type GALC at pH 7.0 or (**b**) at pH 4.5. (**c**) Time resolved distance plot for all four GALC wild type simulation runs at pH 4.5. The donor-acceptor distance of the Thr112:Gln107 hydrogen bond was measured. The green dashed lines indicate the distance values within measured distances are assumed to be hydrogen bonds.

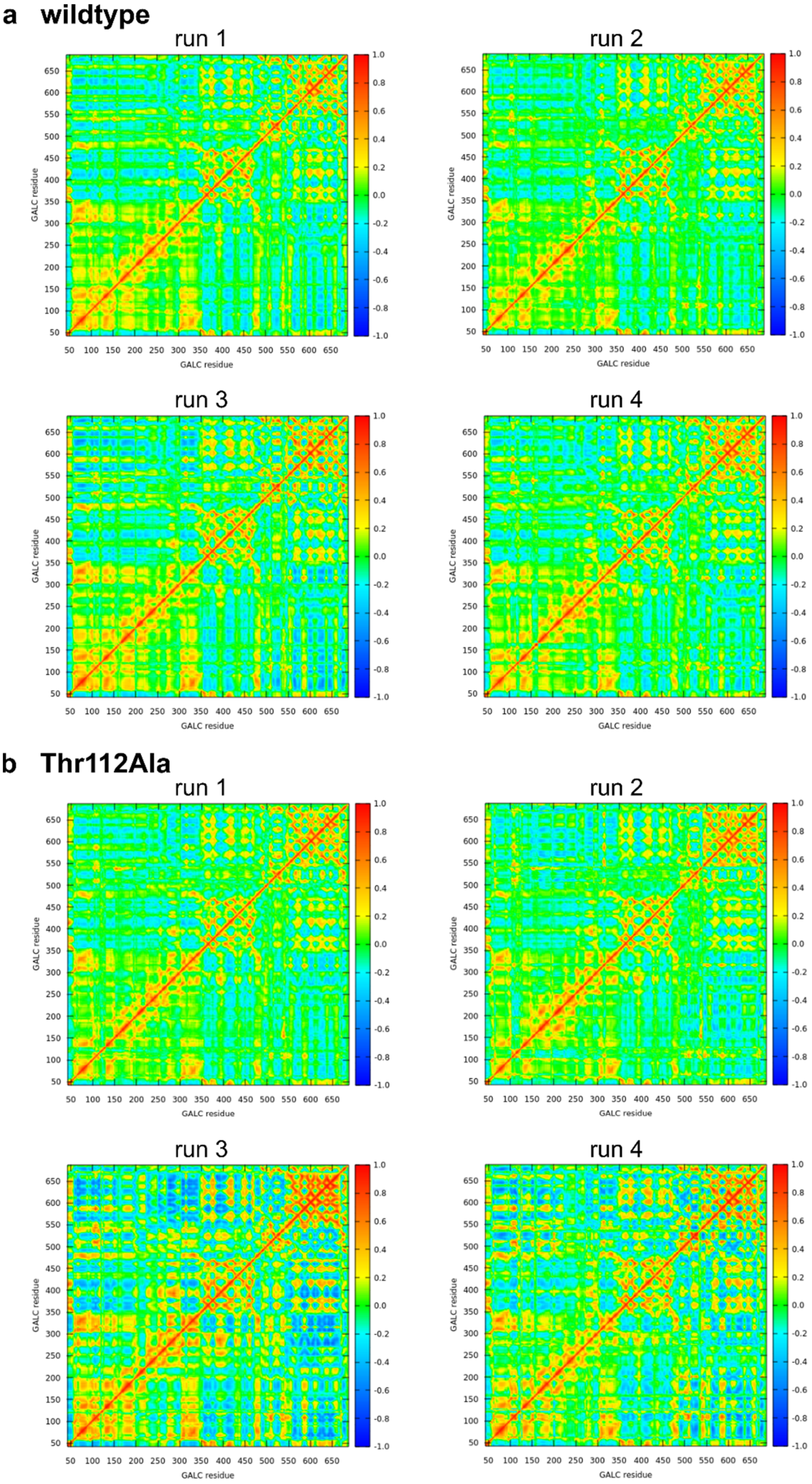

Figure S4. Dynamical cross-correlation maps for wildtype GALC and the Thr112Ala variant at lysosomal pH. Dynamical cross-correlation maps were calculated for Cα atom pairs of GALC based on the four 500-ns MD simulation runs of (**a**) wildtype GALC and (**b**) the Thr112Ala variant at lysosomal pH. Motions of each residue of GALC is color-coded in comparison to all other residues of GALC. Red color indicates high positive correlation between the motions of the corresponding residue pair, while blute color indicates a negative correlation.
